# BDMANGO: An image dataset for identifying the variety of mango based on the mango leaves

**DOI:** 10.1016/j.dib.2024.111241

**Published:** 2024-12-19

**Authors:** Mohammad Manzurul Islam, Md. Jubayer Ahmed, Mahmud Bin Shafi, Aritra Das, Md. Rakibul Hasan, Abdullah Al Rafi, Mohammad Rifat Ahmmad Rashid, Nishat Tasnim Niloy, Md. Sawkat Ali, Abdullahi Chowdhury, Ahmed Abdal Shafi Rasel

**Affiliations:** Department of Computer Science and Engineering, East West University, Aftabnagar, Dhaka, Bangladesh

**Keywords:** Mango leaf, Mango variety, Computer vision, Artificial intelligence, Image classification

## Abstract

In the field of agriculture, particularly within the context of machine learning applications, quality datasets are essential for advancing research and development. To address the challenges of identifying different mango leaf types and recognizing the diverse and unique characteristics of mango varieties in Bangladesh, a comprehensive and publicly accessible dataset titled “BDMANGO” has been created. This dataset includes images essential for research, featuring six mango varieties: Amrapali, Banana, Chaunsa, Fazli, Haribhanga, and Himsagar, which were collected from different locations. The images were captured using the rear cameras of a Google Pixel 6a and an iPhone XR and were stored in 640 × 480 pixels resolution. Both sides of each mango leaf were photographed against white background to accurately reflect real-world scenarios in mango cultivation fields. The white background was specifically chosen to remove noise in image sample, allowing for accurate feature extraction by machine learning algorithms. This will ensure the trained model's efficacy in identifying a specific mango leaf while implemented alongside any segmentation algorithm. Additionally, image augmentation techniques such as rotation, horizontal flip, vertical flip, width shift, height shift, shear range, and zooming were applied to expand the dataset from 837 original images to a total of 6696 images (837 original image and 5859 augmented images). This expansion significantly enhances the dataset's utility for training, testing, and validating machine learning models designed for classifying mango leaf varieties, thereby supporting research efforts in this domain.

Specifications Table

**Table:** 

Subject	Computer Vision and Pattern Recognition, Agriculture Sciences
Specific subject area	Computer Vision, Image Processing, Image Classification, Machine Learning
Type of data	Image (Original, Augmented)
Data collection	• Mango varieties: Amrapali, Banana, Chaunsa, Fazli, Haribhanga, Himsagar. • Collection season: Peak summer season when trees are in full leaf. • Photography setup: • Google Pixel 6A: 12.2 MP (f/1.7, 27 mm, (wide), 1/2.55″, 1.4 µm, dual pixel PDAF, OIS). • iPhone XR: 12 MP (f/1.8, 26 mm (wide), 1/2.55″, 1.4 µm, PDAF, OIS). • Image background: White background was used for reducing noise. • Images captured: 837 original images. Dataset Augmentation: Expanded to 5859 images through augmentation techniques, resulting in a total of 6696 images.
Data source location	1 Fakirkhali Govt. Primary School (Latitude: 23.780990480503522, Longitude: 90.46967067116383) 2 Gouranagar Mosque (Latitude: 23.76038982054776, Longitude:90.472443227771*)*
Data accessibility	Repository name: Mendeley Data Data identification number: 10.17632/nnh69sng8p.5 Direct URL to data: https://data.mendeley.com/datasets/nnh69sng8p/5 Researchers and enthusiasts can access the URL and find the original and augmented image folders. They can directly download the image and proceed with their research.
Related research article	None

## 1 Value of the Data

• This is a unique dataset of mango leaf images based on the mango variety of Bangladesh, a leading mango-growing country. The dataset comprises 837 images, all captured using two mobile phone cameras from various locations and subsequently categorized manually. Through augmentation, the dataset was expanded to include a total of 6696 images. The dataset includes images of both the front and back sides of mango leaves. This dual-side capture provides a more comprehensive representation of the leaf structure and features, enhancing the dataset's utility for accurate classification and analysis.
• Mango farming industry can utilize this dataset and develop autonomous AI-based young mango tree sorting system based on the type of mangoes. This can expeditated relevant supply chain system that procure and deliver young mango trees.
• The dataset is well-organized into six sub-folders, each representing one of the mango varieties. This structured categorization facilitates easy access and efficient use for researchers aiming to classify and study different mango leaf types.
• The dataset is freely available for public download, allowing researchers from around the world to use it for training, testing, and validating their machine learning models. This accessibility promotes collaboration and accelerates research in mango leaf classification. Although focused on mango varieties from Bangladesh, the dataset's size and diversity make it suitable for machine learning applications. Researchers can leverage this dataset to classify mango leaf varieties, promoting broader applicability and research potential.

## 2 Background

Mango production plays a crucial role in the agricultural landscape of Bangladesh, contributing significantly to its economic growth, particularly during the summer season (March–August). Bangladesh ranks as the 9th highest mango-producing country in the world [4,10]. Among the more than fifty different fruits grown in Bangladesh, mango holds a special place [1]. In Rajshahi and Chapainwabganj, 23.3 % and 26.7 % of growers, respectively, have over 25 years of experience in mango cultivation [8]. Mangoes of superior varieties, including Fazli, Langra, Gopalbhog, Himsagar, Khirsapati, Ashhwina, Haribhanga, Rupali, Chaunsa, and Khisanbhog, are among those cultivated in this region [13]. To create our dataset, samples were taken from six different varieties of mango leaves to ensure a varied representation of sizes, shapes, and textures [2]. While traditionally mango varieties in Bangladesh are identified by horticulturists and agricultural experts through visual inspection, recent technological advancements have paved the way for more efficient and accurate methods of classification using machine learning. These advanced techniques have shown the potential to classify different types of mango leaves with a high degree of accuracy, provided that sufficient and high-quality data is available for training the models.

Mango is one of the most popular fruits in the world, ranking among the top five or six fruits in terms of production, with a global annual yield of about 50 million metric tons [11]. Bangladesh is renowned for its diverse mango varieties, with each type exhibiting unique characteristics. In literature, machine learning (ML) and deep learning (DL) based approaches have demonstrated encouraging results in classifying grapevine cultivars from leaf images [16]. Leaf image-based methods, like the multi-feature combined cultivar identification system (MFCIS), have shown potential in cultivar identification by combining morphological features with deep learning techniques [17]. These methods require benchmark datasets to validate the efficacy of ML/DL algorithms. The dataset in [18] includes 768 images from 32 types of mango leaves. However, low number of images (24 images per class) in each category limits its usefulness in training comprehensive machine learning models. Although the dataset by Hena et al. [19] contains more images in total, it does not provide any sample for 5 out of 6 classes present in our BDMANGO dataset. This study addresses this gap by providing a standard, ready-to-use, and publicly available dataset specifically designed for the classification of mango leaf varieties. This dataset is intended to support researchers in developing and testing machine learning models that can accurately classify the various mango varieties cultivated in Bangladesh, thereby contributing significantly to the field of agricultural informatics and mango varietal research. Table 1 compares our dataset with existing mango leaf datasets.

**Table 1:** Comparison with existing datasets.

Mango Type	Our Dataset (BDMANGO)		Paper [18]	Paper [19]
	Original	Augmented		
Amrapali Mango	120	840	96	x
Banana Mango	167	1169	x	x
Chaunsa Mango	150	1050	48	x
Fazli Mango	160	1120	x	339
Haribhanga Mango	120	840	x	x
Himsagar Mango	120	840	x	x

## 3 Data Description

**Fig. 1:**
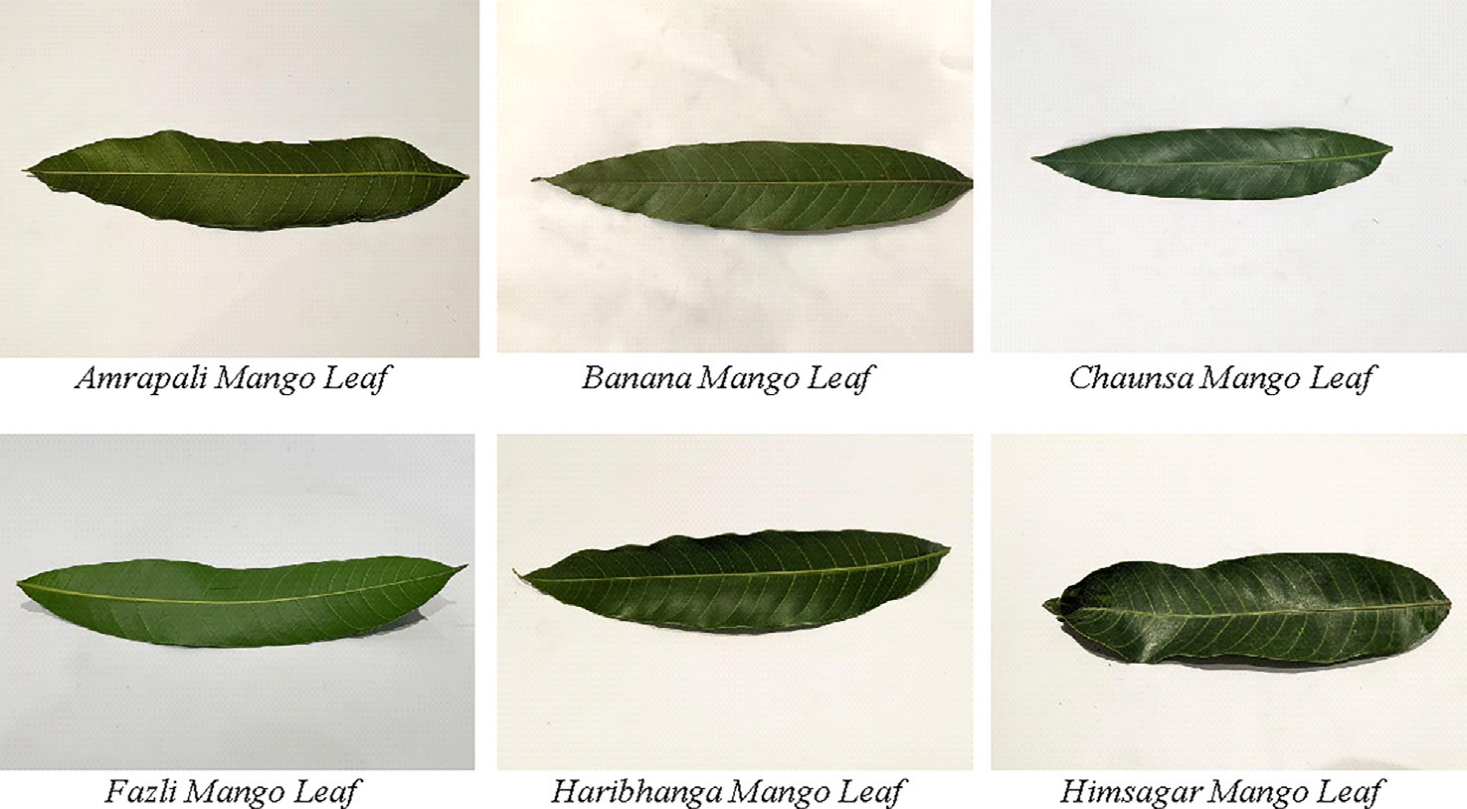
Sample of six different types of mango leaf image sample (original) from image dataset.

The dataset is particularly valuable for researchers aiming to classify mango leaf images using machine learning models. It includes six different types of mango varieties, providing a diverse collection that helps in creating accurate classification models. The mango leaf dataset includes the following varieties: Amrapali, Himsagar, Chausa, Banana, Fazli, and Haribhanga mango leaves. Fig. 1 displays the sample images (original) from the dataset.

The first sample shows the Amrapali mango leaf. Amrapali mangoes are known for their sweet flavor and have vibrant green leaves. The second sample shows the Banana mango leaf. Banana mangoes have long, narrow leaves that are light green. The third one shows the Chausa mango leaf whose mangoes are juicy with broad, glossy leaves. The fourth leaf is the Fazli mango leaf. Fazli mangoes are large and ripen late, with broad, dark green leaves. The fifth one shows the Haribhanga mango leaf. Haribhanga mangoes are known for their unique taste and smell, with oval-shaped, dark green leaves. The last sample the Himsagar mango leaf. Himsagar mangoes are very sweet, and their leaves are a rich green color. A short description of the dataset is provided in Table 2.

**Table 2:** Mango leaf dataset summary.

Type of data	640 * 480 mango leaf images
Data Format	JPG
No of images	6696 images (837 original images + 5859 augmented images)
Mango varieties considered	Total six mango varieties, namely Amrapali, Banana, Chaunsa, Fazli, Haribhanga, and Himsagar.
Number of classes	Total six classes (Amrapali Mango, Banana Mango, Chaunsa Mango, Fazli Mango, Haribhanga Mango and Himsagar Mango.)
Distribution of original images	Amrapali Mango, Haribhanga Mango, Himsagar Mango contains 120 images, Banana Mango contains 167 images, Chaunsa and Fazli mango contains 150 and 160 images respectively.
Distribution of Augmented images	Amrapali Mango, Haribhanga Mango, Himsagar Mango contains 840 images, Banana Mango contains 1169 images, Chaunsa and Fazli Mango contains 1050 and 1120 images, respectively.
How data are acquired	After collecting the leaves, the samples were photographed on a white background using two mobile phones, capturing both sides of each leaf to ensure detailed representation.
Data source locations	Fakirkhali Govt. Primary School and Gouranagar Mosque.
Where applicable	Suitable for mango leaf variety classification.

To reduce any differences in image quality, both devices were used in similar conditions, including consistent lighting, and a plain white background to capture the images, ensuring that the leaf was the focus of the image and that the background did not introduce any unwanted variability or noise. Each image was manually reviewed for focus, exposure, and clarity. Blurry or improperly lit images were discarded before further processing. All images were resized to 640 × 480 pixels to ensure uniformity across the dataset.

**Fig. 2:**
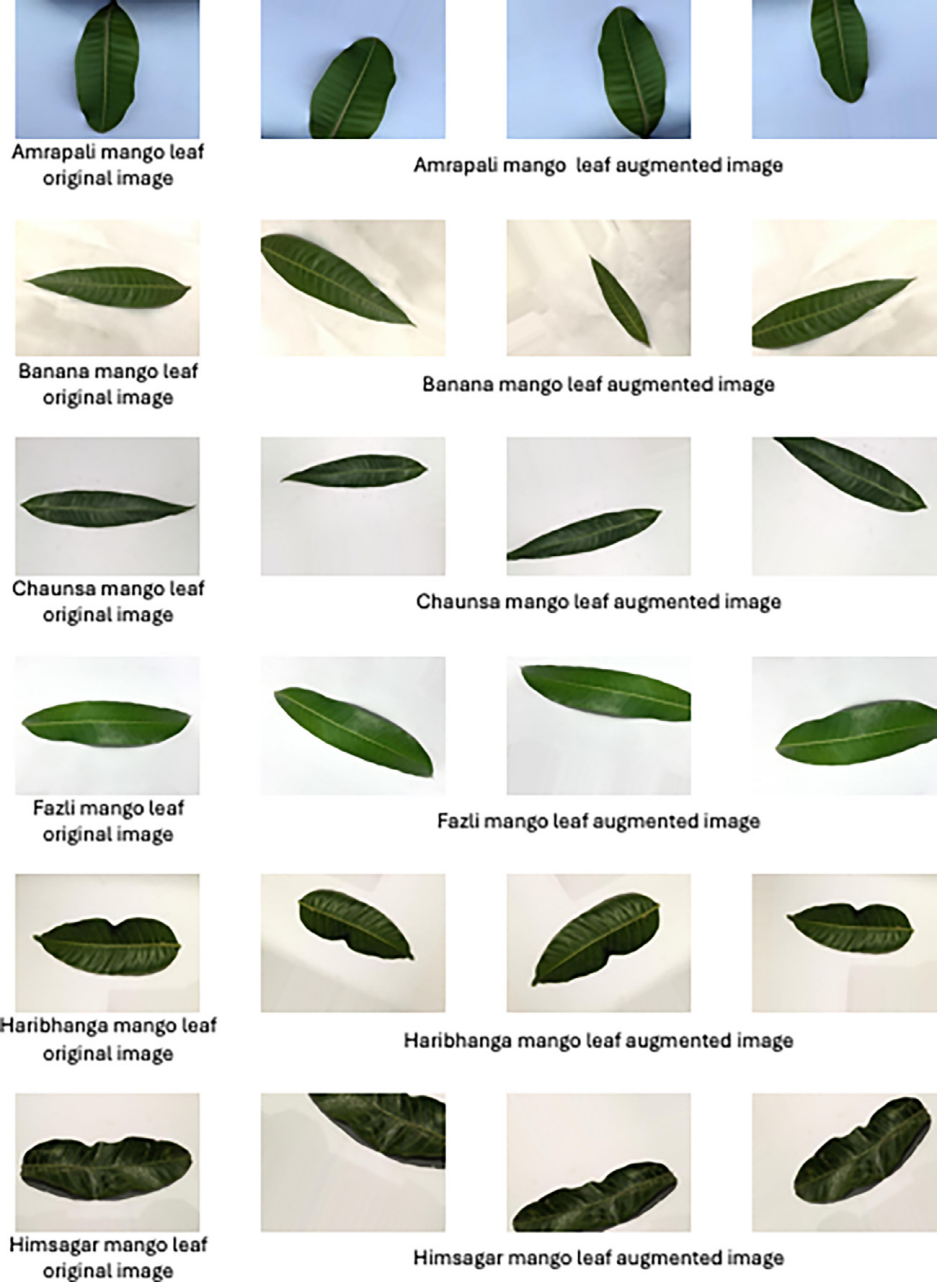
Example of original and corresponding augmented images of different classes.

We augmented the original images and further enhanced our dataset to a total of 6696 images (837 original images + 5859 augmented images). Details of augmentation techniques and statistics are further discussed in “Image Augmentation” section. Fig. 2 illustrates the examples of each image augmentation method.

**Fig. 3:**
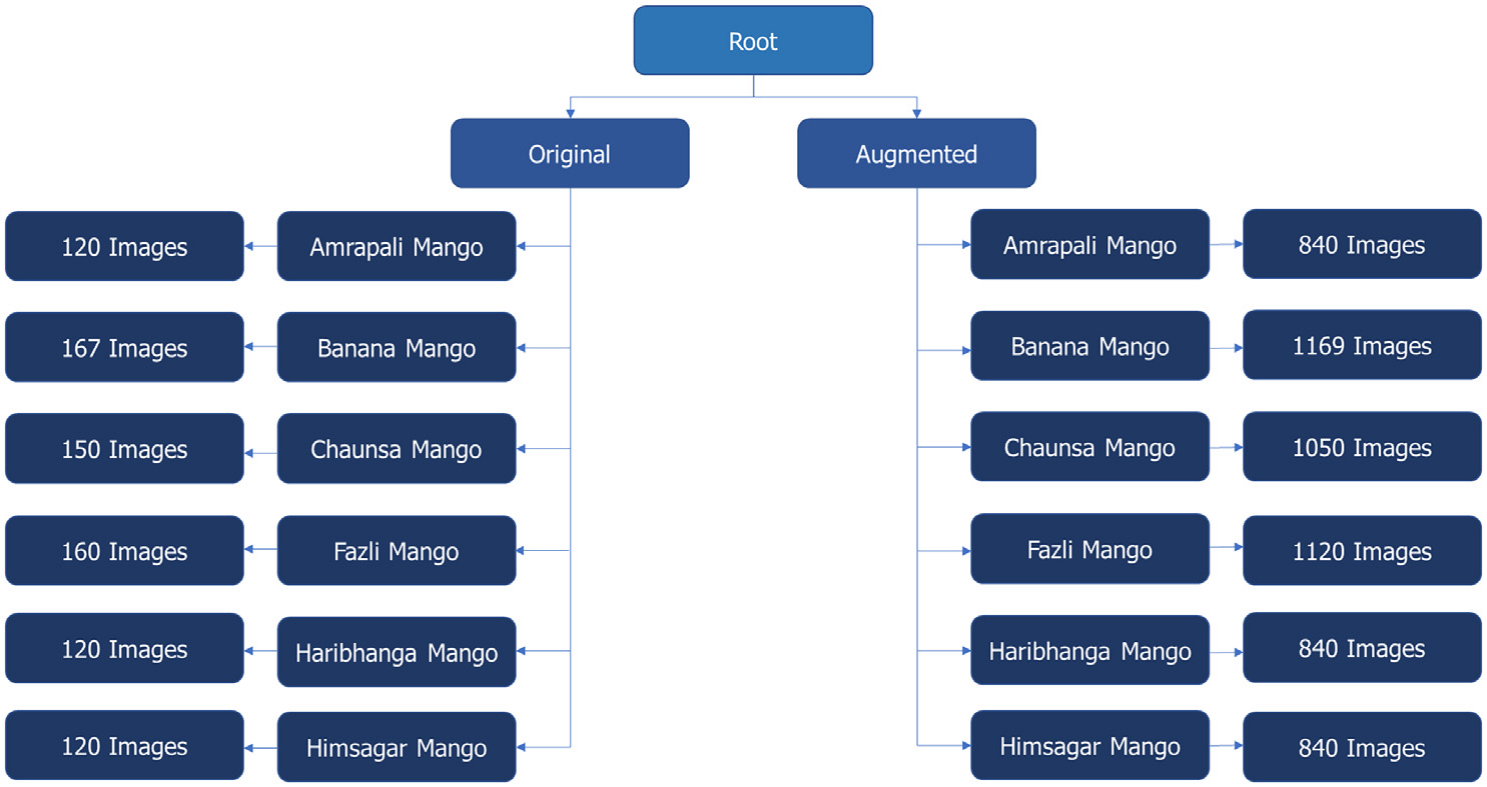
Structure of the directory of the dataset.

The root directory in the repository consists of two main folders: “Original” and “Augmented.” Each of these folders contains six sub-folders named after the mango varieties: Amrapali Mango, Banana Mango, Chaunsa Mango, Fazli Mango, Haribhanga Mango, and Himsagar Mango. These sub-folders contain the respective images. In the “Original” directory, each sub-folder contains the initial set of images, while in the “Augmented” directory, each sub-folder contains the augmented versions of these images. Fig. 3 shows how the folders and sub-folders are organized for the images.

## 4 Experimental Design, Materials and Methods

The key to the successful application of machine learning is the quality of the dataset used. Thus, there is a strong correlation between the quality of the dataset and the performance of the machine learning system [6,7,9]. Machine learning algorithms work by extracting hidden patterns from samples during the training phase and using these patterns to predict the correct classification of new data. Therefore, the performance of these algorithms is closely tied to the quality of the dataset, which can be evaluated based on factors such as its size, balance in data distribution across classes, the absence of noise in the data. Various machine learning models have already been used to classify different mango varieties [5,13]. A high-quality dataset must accurately represent real-world conditions to maximize the effectiveness of the machine learning models that utilize it.

To ensure diversity, we selected six different types of mangoes and collected corresponding leaves. All images were captured under uniform conditions, including a white background for consistency, positioning the leaves centrally, and photographing both sides of each leaf. To add further diversity, we augmented the images by varying leaf position by applying the rotation angle ranging from −30 to 30° on each leaf image. To avoid bias, we ensured that each original category contained at least 120 images, with counts ranging from 120 to 167. This approach maintains balanced representation across categories, with no category having a significantly higher or lower count than others. In the augmented dataset, the image count ranges from 840 to 1169, with a difference of 329 images, maintaining relative balance without introducing substantial bias.

Mango leaf data acquisition process are as follows:

1. Various types of mangoes commonly found in Bangladesh, focusing on their distinct leaf characteristics has been studied.
2. Different key locations have been identified and selected for data collection, ensuring that they were representative of the different mango varieties.
3. Leaves were carefully collected from the trees in the selected locations, ensuring that both sides of the leaves in good condition. After collecting the leaves, those were brought to a controlled environment where images were taken against a white background. The white background was chosen to ensure consistency and clarity in the images, facilitating more precise feature extraction by the machine learning model. A uniform background minimizes distractions and eliminates environmental noise, enabling the model to focus on specific leaf characteristics, such as shape, texture, and patterns.
4. The collected images were carefully curated and pre-processed for augmentation by organizing them into six sub-folders based on mango varieties, standardizing image sizes, enhancing quality, and correcting any background noise or inconsistencies.
5. Applying image augmentation strategies to increase the dataset, boosting its variety and quantity to improve the effectiveness of model training.

**Fig. 4:**
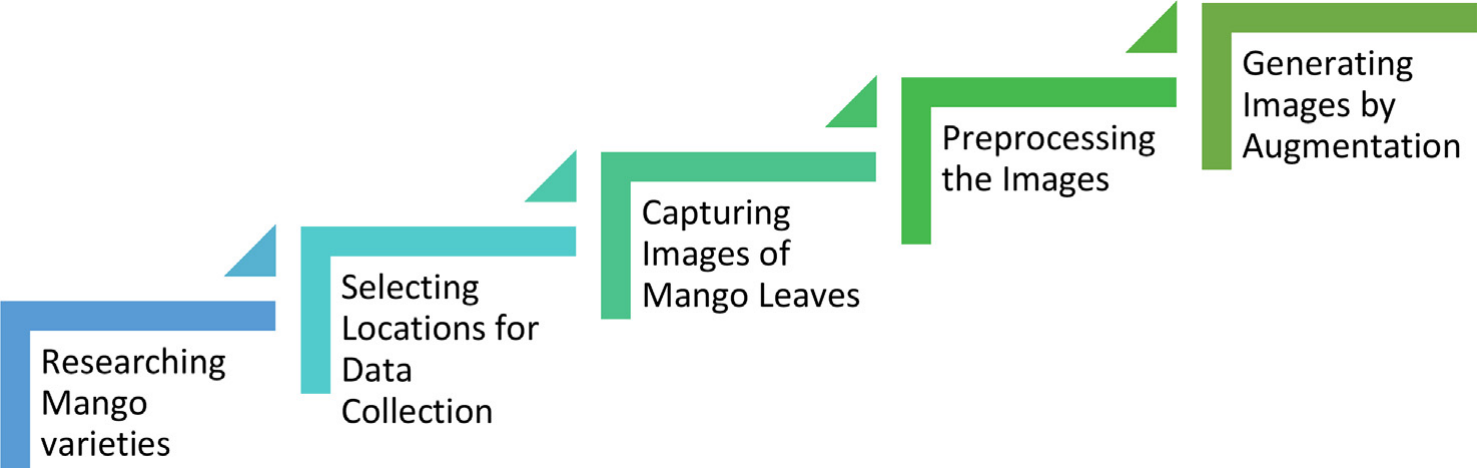
Flowchart of data preparation steps.

Fig. 4 shows a quick glance at the data preparation steps.

### 4.1 Studying the common mango varieties in Bangladesh

Amrapali is the first commercialized mango hybrid, known for its distinctly dwarf stature and regular, prolific bearing. It is precocious in nature and is highly suitable for high-density planting, making it a popular choice among mango farmers [12]. Haribhanga is a traditional commercial cultivar of mango, renowned for its fibreless and very fleshy texture. It is a highly sought-after variety due to its excellent taste and the fact that the fruit typically weighs between 300 and 400 g s, making it an ideal choice for consumers [3]. Chaunsa is highly regarded for its rich, aromatic flavor and luscious texture. It is a late-season mango, often harvested at the peak of the mango season, and is known for its golden-yellow skin. Fazli Mango is known for its large size and late season harvesting. It has a unique flavour profile, with a perfect balance of sweetness.

### 4.2 Selecting the mango orchards

To collect data, suitable mango orchards were selected from various areas within Bangladesh. The chosen locations are the Fakirkhali Government Primary School Orchard (Latitude 23.780990480503522, Longitude 90.46967067116383) and the Gouranagar Mosque Orchard (Latitude 23.76038982054776, Longitude 90.472443227771). These sites feature a diverse range of mango varieties, which helped create a comprehensive dataset. From the leaf samples at the Fakirkhali Government Primary School Orchard, 160 images were collected. In contrast, 677 images were captured from the samples obtained at the Gouranagar Mosque Orchard.

### 4.3 Collecting and capturing the image

To build the dataset for this research, in May 2024, two locations with rich collection of the selected variety were chosen. The purpose was to collect leaves from six different mango varieties: Amrapali, Banana Mango, Chaunsa, Fazli Mango, Haribhanga, and Himsagar. These specific varieties were selected to represent a broad spectrum of mango species in the region. The collection process began by identifying healthy trees of each variety. Leaves were carefully plucked from these trees to ensure a diverse set of samples. To maintain the quality of the collected leaves for image capture, they were promptly stored in cool, shaded conditions immediately following collection, minimizing any deterioration before photographing. Furthermore, samples were gathered from multiple areas across the orchard to capture a realistic range of natural variations in leaf size, shape, and overall health. By selecting leaves from different trees and at various times throughout the day, we ensured that the dataset would represent a broad spectrum of leaf characteristics typically present within the orchard. After gathering the leaves from the field, the next process was to capture the images in a controlled environment. Each leaf was photographed individually by placing it on a white background. Both the front and back sides of the leaves were captured to provide a comprehensive view. Photos were taken using the rear cameras of two smartphones: a Google Pixel 6A with a 12.2 MP (f/1.7, 27 mm) camera and an iPhone XR with a 12 MP (f/1.8, 26 mm) camera. The photo sessions were conducted under controlled indoor lighting to mimic natural daylight conditions, ensuring consistent image quality. Since the leaves were collected and photographed on the same day, there was no delay in capturing the images, which helped preserve their natural texture and appearance. The temperature and humidity during the image capture process were typical of indoor conditions in the month of May, ensuring no external environmental factors affected the leaves' appearance. This method resulted in a total of 837 images, distributed across the six mango leaf varieties.

### 4.4 Processing the dataset images

After capturing the images, these were distributed into six separate folders, each named according to the mango variety. To ensure that the images meet the requirements of machine learning models, each photo was saved to 640 × 480 pixels in JPG format. This step standardizes the images for better visualization and consistency.

### 4.5 Image augmentation

**Fig. 5:**
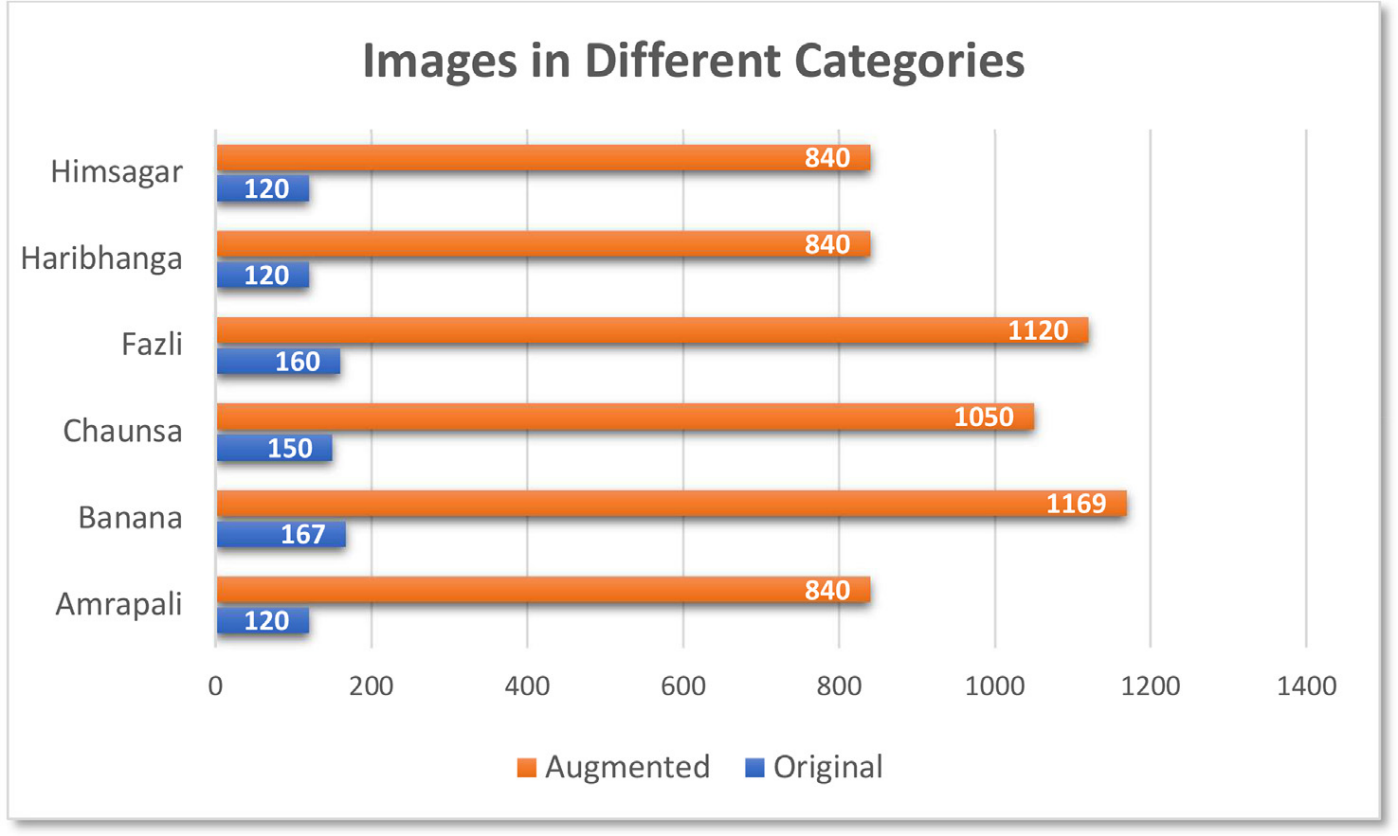
Total Number of Mango Leaves in the dataset.

From 837 original images, 5859 additional images were generation adopting a variety of image augmentation techniques, including rotation, horizontal flip, vertical flip, width shift, height shift, shear range, and zooming. As a result, a total of 6696 images were included in the dataset. The statistics of the images are illustrated in Fig. 5.

The following augmentation methods were selected to increase dataset variability and enhance the model's robustness by enabling it to recognize leaves in different orientations and conditions, thereby improving its generalizability to new, unseen data. In machine learning tasks, especially image classification, having a diverse and varied dataset is crucial to prevent overfitting, where a model performs well on the training data but poorly on new data. Augmentations act as a form of regularization by exposing the model to a broader range of training examples, leading to improved performance and higher accuracy in real-world applications. This is particularly important in tasks like leaf classification, where subtle changes in leaf orientation or appearance are common, and the model needs to be trained to handle these variations. Below is a detailed summary of the augmentation processes:

1. ***Rotation:*** Adjusting the orientation of an image by tilting it within a specified angle range, such as [−30, 30] degrees, has been found to be effective in simulating different viewpoints. This technique helps in generalizing the model's ability to recognize objects regardless of their rotation. When images are rotated during augmentation, blank spaces appear around the edges, creating gaps that can introduce artifacts or distortions in the dataset. These spaces are filled with a uniform color to ensure consistency, avoid unwanted artifacts, and prevent model bias [15]. In our case, this uniform color was chosen to be ‘white’ to ensure reliable training without skewing color distributions.
2. ***Horizontal and Vertical Flip:*** Flipping images along the horizontal or vertical axis provides mirrored versions of the original images. This method is useful for enhancing the model's ability to recognize objects from different orientations. By reversing the complete rows and columns of pixels, these flips generate alternative perspectives that can help improve the model's recognition accuracy [15].
3. ***Width and Height Shift:*** Horizontal and vertical shifts are controlled by parameters that define the maximum displacement applied to the image. Typically, a horizontal shift up to 20 % of the image's total width and a vertical shift up to 20 % of the image's total height are used to simulate changes in the object's position within the frame [14].
4. ***Zoom Range:*** Zoom augmentation involves adjusting the scale of the image by a certain percentage. A maximum zoom of 20 % of the image's size is commonly applied, allowing for variations in the perceived distance from the object [14].
5. ***Fill Mode:*** This method specifies how pixels lost during the augmentation process are handled. Typically, gaps created during transformations like rotation or shifting are filled with the closest pixel value to maintain the continuity of the image [14].

After completing these augmentation techniques, a total of 6696 images have been included in the final dataset.

## Limitations

• The dataset was constructed using mango leaves collected from Dhaka, Bangladesh, which may not fully represent the diversity of mango trees across other mango-growing regions.
• This dataset includes six specific mango varieties. However, there exists other mango varieties that are not represented in this dataset. However, our selected mango types are the most popular breed of mangoes that capture a major share of mango production and consumption in Bangladesh.
• Since the dataset focuses exclusively on leaves, the model may not generalize to images that include other plant parts (e.g., branches, fruits) on major part of captured image, which might be present in some practical applications. However, careful image collection by the application software may solve this issue.

## Ethics Statement

The ethical prerequisites for publishing in Data in Brief have been thoroughly reviewed and adhered to. It is hereby validated that the present study does not entail the participation of animal experimentation, human subjects, or the utilization of any data acquired from social media platforms.

## Credit Author Statement

**Mohammad Manzurul Islam:** Conceptualization, Methodology, Supervision, Project Administration. **Md. Jubayer Ahmed:** Investigation, Data curation, Data collection, **Mahmud Bin Shafi:** Data collection, Writing - original draft, review & editing. **Aritra Das:** Methodology, Visualization, Investigation, Data curation. **Md. Rakibul Hasan:** Data description, Writing - original draft, review & editing. **Md. Abdullah Al Rafi:** Writing – review & editing. **Mohammad Rifat Ahmmad Rashid:** Writing - review & editing. **Nishat Tasnim Niloy:** Writing – review & editing, Validation, Investigation. **Md. Sawkat Ali**: Supervision. **Abdullahi Chowdhury:** Supervision. **Ahmed Abdal Shafi Rasel:** Methodology.

## Data Availability

Mendeley DataFind Research Data My Data MM Image Dataset of Bangladeshi Mango Leaf (Original data).

## References

[1] Abuhena M., Rashid J.A., Azad A.K. (2024). Analysis of genetic variability among regional mango varieties grown in Rajshahi district using RAPD markers. Ecol. Genet. Genom..

[2] Ara R., Motalab M., Uddin M., Fakhruddin A., Saha B. (2014). Nutritional evaluation of different mango varieties available in Bangladesh. Int Food Res J.

[3] Begum L., Rahman A., Rahman H., Islam T., Ahmed M., Arfin S., Akter N. (2023). Changes of postharvest nutritional quality and antioxidant enzymes in ‘Haribhanga’ mango by aloe vera gel with chitosan and coconut oil coating during ambient storage. J. Horticult. Res..

[4] Big, A. (n.d.). *World mango and guava production by country*. Retrieved August 30, 2024, from AtlasBig: https://www.atlasbig.com/en-gb/countries-by-mango-guava-production.

[5] Dong Z., Wang J., Sun P. (2024). Mango variety classification based on convolutional neural network with attention mechanism and near-infrared spectroscopy. Food Measure.

[6] Fenza G.M., Gallo M., Loia V., Orciuoli F., Herrera-Viedma E (2021). Data set quality in machine learning: consistency measure based on group decision making. Appl. Soft Comput..

[7] Gupta N., Mujumdar S., Patel H., Masuda S., Panwar N., Bandyopadhyay S., Munigala V. (2021). Proceedings of the 27th ACM SIGKDD Conference on Knowledge Discovery & Data.

[8] Hoque A., Bhuiyan M.S., Ahiduzzaman M., Akhter S., Hossain M.S. (2023). Scenario of mango marketing by farmers in selected areas of Bangladesh. Res. Rev.: J. Crop Sci. Technol..

[9] Jain A., Patel H., Nagalapatti L (2020). Proceedings of the 26th ACM SIGKDD International Conference on Knowledge Discovery & Data Mining.

[10] Kobra K., Hossain M., Talukder M., Bhuyan M. (2012). Performance of twelve mango cultivars grown in different agro-ecological zones of Bangladesh. Bangl. J. Agricult. Resour..

[11] M. S. (2024). *Fruit: world production by type 2022*. Retrieved August 30, 2024, from Statista: https://www.statista.com/statistics/264001/worldwide-production-of-fruit-by-variety/.

[12] Meena N.K., Asrey R. (2018). Tree age affects postharvest attributes and mineral content in amrapali mango (Mangifera indica) fruits. Horticult. Plant J..

[13] Shahriar M., K., R. M., S., M. S (2023). 26th International Conference on Computer and Information Technology (ICCIT).

[14] Shorten C., Khoshgoftaar T.M. (2019). A survey on image data augmentation for deep learning. J. Big Data.

[15] Xu M., Yoon S., Fuentes A., Park D.S. (2023). A comprehensive survey of image augmentation techniques for deep learning. Pattern Recognit.

[16] Vélez S., Rubio J.A., Vacas R., Barajas E. (2024). Digital ampelography: deep learning (CNN) using Keras to identify grapevine cultivars. Acta Hortic..

[17] Zhang Y. (2021). MFCIS: an automatic leaf-based identification pipeline for plant cultivars using deep learning and persistent homology. Hortic. Res..

[18] “Mango Leaf Species (Indian),” Kaggle, Mar. 23, 2020. https://www.kaggle.com/datasets/dalipkamboj/mango-leaf-species-indian.

[19] Hena H., Marouf A.A., Sultana R. (2019). MangoLDB: a dataset of mango leaves RGB, binary and gray-scale image. Int. J. Innovat. Technol. Explor. Eng.(IJITEE).

